# *Opuntia ficus-indica* (L.) Mill.: A Multi-Benefit Potential to Be Exploited

**DOI:** 10.3390/molecules26040951

**Published:** 2021-02-11

**Authors:** Mafalda Alexandra Silva, Tânia Gonçalves Albuquerque, Paula Pereira, Renata Ramalho, Filipa Vicente, Maria Beatriz P. P. Oliveira, Helena S. Costa

**Affiliations:** 1 Departamento de Alimentação e Nutrição, Instituto Nacional de Saúde Doutor Ricardo Jorge, I.P., Av. Padre Cruz, 1649-016 Lisboa, Portugal; mafalda.silva@insa.min-saude.pt (M.A.S.); tania.g.alb@gmail.com (T.G.A.); 2 REQUIMTE-LAQV/Faculdade de Farmácia da Universidade do Porto, Rua de Jorge Viterbo Ferreira, n°. 228, 4050-313 Porto, Portugal; beatoliv@ff.up.pt; 3 Instituto Universitário Egas Moniz, Campus Universitário, Quinta da Granja, Monte de Caparica, 2829-511 Caparica, Portugal; pereira.paula@gmail.com (P.P.); renata.c.ramalho@gmail.com (R.R.); filipavicente@hotmail.com (F.V.); 4 CiiEM—Centro de Investigação Interdisciplinar Egas Moniz, Campus Universitário, Quinta da Granja, Monte de Caparica, 2829-511 Caparica, Portugal; 5 GENA—Grupo de Estudos de Nutrição Aplicada, Campus Universitário, Quinta da Granja, Monte de Caparica, 2829-511 Caparica, Portugal

**Keywords:** *Opuntia ficus-indica* (L.) Mill., byproducts, nutritional composition, bioactive compounds, biological activities, food sustainability, food waste

## Abstract

Consumer interest in foods with enhanced nutritional quality has increased in recent years. The nutritional and bioactive characterization of fruits and their byproducts, as well as their use in the formulation of new food products, is advisable, contributing to decrease the global concerns related to food waste and food security. Moreover, the compounds present in these raw materials and the study of their biological properties can promote health and help to prevent some chronic diseases. *Opuntia ficus-indica* (L.) Mill. (prickly pear) is a plant that grows wild in the arid and semi-arid regions of the world, being a food source for ones and a potential for others, but not properly valued. This paper carries out an exhaustive review of the scientific literature on the nutritional composition and bioactive compounds of prickly pear and its constituents, as well as its main biological activities and applications. It is a good source of dietary fiber, vitamins and bioactive compounds. Many of its natural compounds have interesting biological activities such as anti-inflammatory, hypoglycemic and antimicrobial. The antioxidant power of prickly pear makes it a good candidate as an ingredient of new food products with fascinating properties for health promotion and/or to be used as natural extracts for food, pharmaceutic or cosmetic applications. In addition, it could be a key player in food security in many arid and semi-arid regions of the world, where there are often no more plants.

## 1. Introduction

Nowadays, health professionals, as well as consumers, are increasingly aware of food and its potential health benefits. There is a growing interest in the nutritional composition and bioactive compounds of foodstuffs as well as the benefits for disease prevention. On the other hand, the reduction of wastes discarded by the food industry has also become one of the main objectives of the European Commission. Thus, the European Commission has supported several actions against food waste [1]. One of the appointed solutions involves the use of byproducts in the formulation of new products, and their study in terms of nutritional composition and bioactive compounds becomes extremely important.

Green chemistry is a quite novel concept that aims to reduce hazards across all the life-cycle stages, to reduce environmental impact. Moreover, it should be an economically profitable approach. According to green chemistry’s twelve principles, it is better to prevent waste generation than to treat or clean it up. However, in the food industry, it is quite inevitable to prevent byproducts generation, which most of the time is treated as waste. Therefore, it is particularly important to valorize this waste by means of green technologies (more innocuous solvents and auxiliaries), which allow to the extraction of bioactive compounds that can be further used, for example, to develop functional foods and/or to obtain added value extracts [2].

*Opuntia ficus-indica* (L.) Mill., also known as prickly pear, is a plant originating from Mexico and belonging to the Cactaceae family. This plant can also be found in all American hemispheres and grows worldwide, such as Africa, Australia and the Mediterranean basin [3,4]. *O. ficus-indica* is gaining interest across the world because it can grow where no other crops are able to do that. This the case of some countries, such as Ethiopia, where it is the only crop that can be relied on [5]. In addition to its use in the diet, prickly pear is also used for healthcare due to its high content of polyphenols and antioxidant, anti-inflammatory and anxiolytic properties [6,7,8,9]. *O. ficus-indica* is a multipurpose crop, not only to provide food and feed but as a source of bioactive compounds with promoting health properties. This review aims to provide an overview of the nutritional composition and bioactive compounds of *O. ficus-indica* (L.) Mill., its constituents, as well as its principal biological activities and the main applications described for this plant. This review exploits the potential of prickly pear and its constituents to produce new food ingredients with health promotion properties and/or to be a source of extracts for application in food, pharmaceutics or cosmetic industries.

## 2. Chemical Composition

The chemical composition of prickly pear and cladodes depends on many factors: species, cultivar, or variety; environmental factors, such as the climatic and edaphic conditions, crop management, including fertilization and postharvest treatment and maturity status [5,10].

### 2.1. Nutritional Composition

The nutritional composition of the different parts of *O. ficus-indica* is resumed in Table 1. The prickly pear pulp is essentially characterized by a high content of water and sugars [11,12,13,14,15,16].

A comparative study of the nutritional composition of *Opuntia dillenii* and *O. ficus-indica* pulp was performed by Medina et al. [15], and they observed that *O. ficus-indica* had a higher protein content (0.90 ± 0.26 g/100 g) than *O. dillenii* (0.52 ± 0.12 g/100 g). The nutritional composition of the pulp of nine different cultivars of *O. ficus-indica* was evaluated, and the Ait Baamrane cultivar has a higher content of total sugars and protein than the Alkalaa cultivar [13]. Albuquerque et al. [17] determined the nutritional composition of the pulp of *Annona cherimola* Mill., another exotic fruit. If we compare the moisture of the pulps of *O. ficus- indica* and *A. cherimola*, it is found that the pulp of *O. ficus-indica* has a higher water and fat content, 88.47 g/100 g and 0.50 g/100 g, respectively [12,13,14,15,17].

Concerning prickly pear byproducts, seeds are a good source of fiber and protein, showing higher levels than the prickly pear peel [14,25]. Salim et al. [14] analyzed the pulp, peel, and seeds of *O. ficus-indica*. They obtained higher levels of protein and fat for seeds (3.67 ± 0.01 and 3.00 ± 0.17 g/100 g, respectively) than for peel (0.14 ± 0.01 and 0.10 ± 0.01 g/100 g, respectively). The main constituent of *O. ficus-indica* cladodes is water (91–95 g/100 g), but it also contains carbohydrates, fiber and protein in small amounts [10,17,18,19,20].

Hernández-Urbiola et al. [20] studied the nutritional composition of *O. ficus-indica* dehydrated cladodes at different maturation stages of 40 to 135 days. The results showed that the dehydrated cladodes with 60 days contained the highest total fat content and the cladode with 50 days the highest protein content [20].

### 2.2. Minerals

The detailed mineral content of the different parts of *O. ficus-indica* is provided in Table 2. Prickly pear is considered a good source of minerals, namely potassium, magnesium, calcium and sodium [12,15,16,21,28,29]. The presence of magnesium and calcium makes the prickly pear useful in the prevention of osteoporosis [30,31].

Medina et al. [15] observed that the levels of potassium and magnesium were remarkably similar in *O. ficus-indica* green pulp and orange pulp. Dehbi et al. [13] analyzed the *O. ficus-indica* pulp of different cultivars and found that Ait Baamrane was the cultivar that presented higher levels of calcium (31.4 ± 1.02 mg/100 g), magnesium (21.2 ± 0.32 mg/100 g) and phosphorus (31.9 ± 0.21 mg/100 g). On the other hand, potassium was present in higher content in the cultivar Doukkala (220.9 ± 0.99 mg/100 g) [13].

Prickly pear seeds are also rich in minerals, essentially potassium and phosphorus, but other minerals (magnesium, calcium, and sodium) were found [14,22,23,25]. According to the results obtained by Astello-García et al. [26] and Hernández-Urbiola et al. [20], the most abundant minerals present in cladodes are potassium (224–2670 mg/100 g), magnesium (94–1120 mg/100 g) and calcium (177–640 mg/100 g).

Méndez et al. [10] compared cladodes of *O. dillenii* with *O. ficus-indica* and observed that the cladodes of *O. dillenii* present lower levels of calcium (157 ± 26 mg/100 g), zinc (0.251 ± 0.219 mg/100 g) and manganese (0.426 ± 0.387 mg/100 g) than cladodes of *O. ficus-indica* (177 ± 31 mg/100 g; 0.368 ± 0.208 mg/100 g; 0.780 ± 0.241 mg/100 g, respectively). According to data reported by Missaoui et al. [32], *O. ficus-indica* cladodes present higher levels of calcium (7518 ± 162 mg/100 g), followed by sodium (1918 ± 105 mg/100 g), potassium (1684 ± 68 mg/100 g) and magnesium (1380 ± 137 mg/100 g) [32].

The mineral pattern depends on the fruit origin, i.e., the edaphic factors at the site of cultivation, thus explaining the controversial data in the literature [13,34].

### 2.3. Vitamins

Vitamins are nutritionally important constituents of the prickly pear (Table 3). The concentrations of vitamins presented in *O. ficus-indica* vary among the different plant tissues. Prickly pear peel is rich in vitamin E, mainly α-tocopherol (1760 ± 155 mg/100 g of total lipids) [35]. In the pulp, α-tocopherol is also present in greater amounts, compared with other forms of vitamin E [35,36]. Ramadan and Mörsel [37,38] compared the seeds oil with the pulp oil and concluded that the pulp oil has a higher content of vitamin E, namely δ-tocopherol (442 ± 17 mg/100 g of total lipids) [38]. On the other hand, the seed oil had a higher content of γ-tocopherol (33 ± 3 mg/100 g of total lipids) [37]. The prickly pear pulp is a good source of ascorbic acid [39,40], and its content ranged from 17 to 46 mg/100 g [7,15,24,41,42,43,44].

## 3. Bioactive Compounds

Cactus plants are also important sources of bioactive substances and excellent candidates for nutraceutical and functional food preparation. Several authors confirm that prickly pear has a high bioactive potential, being an important source of bioactive compounds and an excellent source of dietary antioxidants, which may have beneficial effects on consumers’ health [45].

### 3.1. Fatty Acids

The fatty acids composition of prickly pear pulp, seed oil and peel are shown in Table 4. The major fatty acids present in prickly pear peels are palmitic acid (C16:0) and linoleic acid (C18:2), according to data reported by Ramadan and Mörsel [35], El-Said et al. [21] and Andreu-Coll et al. [47]. In relation to cladodes, palmitic acid (C16:0), oleic acid (C18:1), linoleic (C18:2) acid and linolenic acid (C18:3) are the major fatty acids [47,48]. The prickly pear seeds oil owns the potential of high-quality edible oil with potential health benefits. The major fatty acids of the seed oil were linoleic (C18:2), oleic (C18:1), palmitic (C16:0), and stearic (C18:0) acids [22,29,35,49,50,51,52]. This shows the interest in the prickly pear as a natural source of edible oil containing essential fatty acids [22,53,54].

In Table 5, the profile of saturated, monounsaturated, and polyunsaturated fatty acids determined in oils extracted from other fruit seeds are presented. If we compare the fatty acid profile of the oil of the prickly pear seeds with the one from oils of other fruit seeds, it is possible to verify that the oil of prickly pear seeds has a fatty acid profile similar to watermelon seeds and grape seeds, with the exception of the saturated fatty acids content, which is superior in the prickly pear. The oil of the seeds of the prickly pear also has a linoleic acid content similar to the content present in blackcurrant and pumpkin seeds oil [55]. The high content of polyunsaturated fatty acids in the oil of prickly pear seeds makes this oil potentially beneficial for health because these fatty acids play a preventive role in cardiovascular diseases. This type of fatty acid is described as having activities to reduce total cholesterol and low-density lipoproteins cholesterol [49].

### 3.2. Amino Acids

The main amino acids (Table 6) present in the prickly pear pulp are serine and proline, and gamma-aminobutyric acid [56,57]. Regarding the amino acids found in prickly pear seeds, the main are arginine (14.62 g/100 g protein) and glutamic acid (20.27 g/100 g protein) [23]. Stintzing et al. [57] evaluated the amino acid content present in pulps of three different cultivars of prickly pear and reported that proline, taurine, and glutamine were present in major quantities. From the results reported, it was also possible to verify that the cultivar Apastillada presented the highest level of proline (1768.7 mg/L) and taurine (572.1 mg/L), while the cultivar Gymno Carpo had the highest glutamine content (574.6 mg/L) [57]. Taurine, semi-essential amino acid, has been considered a cellular protective amino acid. It has been involved in the modulation of the inflammatory response and has demonstrated antioxidant effects [58,59,60].

### 3.3. Sterols

Sterols are essential constituents of cell membranes in animals and plants. Some plant sterols are currently incorporated into foods intended to lower blood cholesterol levels [61]. According to the results reported by Ramadan and Mörsel [37,38], who studied the composition of prickly pear pulp and seeds oils, it was shown that for both oils, the main sterol present was β-sitosterol with 1.12 ± 0.121 and 0.675 ± 0.089 g/100 g, respectively. Campesterol is the second sterol present in larger amounts in both oils (0.874 ± 0.075 g/100 g for pulp oil and 0.166 ± 0.021 g/100 g for seeds oil). β-sitosterol and campesterol are also the sterols present in greater amounts in the prickly pear peel oil with 2.11 ± 0.255 and 0.876 ± 0.231 g/100 g, respectively [35]. Δ7-Avenasterol was only detected in small amounts in seed oil [37], whereas ergosterol was only found in the prickly pear peel [35].

### 3.4. Carotenoids

Carotenoids are important compounds with great benefits for human health, are related to the prevention and reduction of the development of some diseases, such as cardiovascular diseases, cancer and macular degeneration [62].

In cladodes, three carotenoids were quantified: lutein (102 ± 0.07 to 187 ± 0.22 μg/100 g dry weight basis), β-carotene (82 ± 0.22 to 119 ± 0.53 μg/100 g dry weight basis) and β-cryptoxanthin (45 ± 0.60 to 72 ± 0.28 μg/100 g dry weight basis), according to data reported by Jaramillo-Flores et al. [63]. According to Cano et al. [46], the peel had a higher content of total carotenoids than pulp. Moreover, the same authors identified that the main carotenoids in the prickly pear peel of Verdal (orange) variety and Sanguinos (red) variety, respectively, are lutein (767.98 ± 2.20 and 1132.51 ± 1.97 μg/100 g), β-carotene (173.50 ± 2.30 and 200.4 ± 2.83 μg/100 g) and violaxanthin (87.67 ± 3.01 and 93.64 ± 1.87 μg/100 g). Lycopene was found in the peel with a concentration of 45.61 ± 2.68 μg/100 g, whereas in the pulp, only traces were reported. In prickly pear pulp of the varieties described above, the main carotenoids found were lutein (203.90 ± 1.39 and 201.45 ± 2.31 μg/100 g), β-carotene (79.10 ± 2.65 and 37.47 ± 1.67 μg/100 g), violaxanthin (31.95 ± 2.76 and 5.76 ± 0.91 μg/100 g) and zeaxanthin (12.27 ± 1.09 and 14.32 ± 0.83 μg/100 g) [46].

### 3.5. Phenolics

The phenolic group is constituted by many compounds, in particular phenolic acids (hydroxycinnamic acids and hydroxybenzoic acids), flavonoids, lignins and stilbenes. Their antioxidant potential is involved in many health benefits such as prevention of inflammation, cardiovascular dysregulation, and neurodegenerative diseases [8,64]. For example, Abdel-Hameed et al. [33] analyzed the presence of phenolic compounds and identified gallic acid, catechin, quercetin-3-glucose-(1-6)-gallic acid in two cultivars (red and yellow) of prickly pear pulp and peel. The same authors reported that the juices of peels and pulp of the red cultivar had a higher content of total phenolics (1065.15 ± 10.21 and 1152.97 ± 8.49 mg of gallic acid equivalents/100 mL of juice, respectively) than the yellow cultivar. Cano et al. [46] compared the prickly pear pulp with the peel and found a higher content of phenolic compounds present in the peel (630.30 ± 45.14 and 698.37 ± 29.26 mg of gallic acid equivalents/100 g) of Verdal (orange) and Sanguinos (red) varieties, respectively. Guevara-Figueroa et al. [27] detected the presence of gallic acid (6.4 to 23.7 μg/g dry weight basis), coumaric acid (140.8 to 161.8 μg/g dry weight basis), 3,4-dihydroxy-benzoic acid (0.6 to 25.1 μg/g dry weight basis), 4-hydroxybenzoic acid (5.0 to 47.2 μg/g dry weight basis), ferulic acid (5.6 to 347.7 μg/g dry weight basis) and salicylic acid (5.8 to 35.4 μg/g dry weight basis) in the prickly pear cladodes. Chougui et al. [49] analyzed prickly pear seeds of different cultivars and obtained content of total phenolics of 48 ± 1 to 89 ± 5 mg gallic acid equivalents/100 g [49].

Flavonoids are a group of bioactive compounds that exhibit many effects in the protection of the body, and their regular consumption is associated with reduced risk of several chronic diseases. In addition, they have antioxidant, antiviral and antibacterial properties [65]. Kuti [38] identified kaempferol (2.2 ± 0.3 μg/g), quercetin (43.2 ± 2.5 μg/g) and isorhamnetin (24.1 ± 1.0 μg/g) in prickly pear pulp. Guevara-Figueroa et al. [27] identified iso-quercitrin (22.9 to 396.7 μg/g dry weight basis), isorhamnetin-3-*O*-glucoside (45.9 to 322.1 μg/g dry weight basis), nicotiflorin (28.9 to 1465.0 μg/g dry weight basis), rutin (23.6 to 261.7 μg/g dry weight basis) and narcissin (146.9 to 1371.0 μg/g dry weight basis) in cladodes of different *Opuntia* spp. varieties. The prickly pear seeds have contents between 1.5 ± 0.1 and 2.6 ± 0.2 mg quercetin equivalents/100 g, according to the data reported by Abdel-Hameed et al. [33].

### 3.6. Betalains

Betalains are vacuolar pigments composed of a nitrogenous core structure, betalamic acid. Betalains include two classes of compounds: betacyanins (red–violet) and betaxanthins (yellow), and their concentrations vary according to the color of the fruit. They are powerful radical eliminators in chemical systems and act as efficient antioxidants in biological models [46,66,67]. In addition to the phenolic compounds, betalains are particularly important components of the prickly pear [68]. According to the results obtained by Cano et al. [46], prickly pear peel presents higher values of betaxanthins (1.73 ± 0.04 and 2.00 ± 0.15 mg indicaxanthin/100 g) and betacyanins (1.17 ± 0.04 and 2.52 ± 0.10 mg betanin/100 g) for Verdal (orange) and Sanguinos (red) varieties, respectively, than prickly pear pulp.

## 4. Biological Activities

The previously described chemical composition has shown that prickly pear is an important source of vitamins, minerals, fiber, some amino acids and fatty acids with potential benefits for human health. Additionally, several bioactive compounds can also be found in its composition, such as phytosterols, flavonoids and polyphenols. In the following subsections, we describe the evidence on the impact of these compounds in disease prevention and health promotion.

### 4.1. Antioxidant Effects

The antioxidant actions attributed to prickly pear fruit can be due to the presence of several compounds, namely vitamin C, carotenoids, but also polyphenols and flavonoid compounds like quercetin, kaempferol and isorhamnetin [41,69]. Despite some differences within the composition of different cactus structures, it is possible to find some similarities in phytochemicals composition. Boutakiout et al. [70] have suggested that prickly pear cladodes are a good source of natural antioxidant compounds. The authors evaluated antioxidant activity using 2,2-diphenyl-1-picrylhydrazyl (DPPH•), 2,2-azino-bis-3-ethylbenzothiazoline-6-sulfonic acid (ABTS) and ferric reducing antioxidant power (FRAP) assays of prickly pear cladodes. The results obtained varied from 1.78 to 4.10 μmol Trolox equivalents/mL for DPPH•, 12.78 to 23.10 μmol Trolox equivalents/mL for ABTS and 1.74 to 3.33 μmol Trolox equivalents/mL for FRAP. The experimental study conducted by Saad et al. [71] has shown that the *O. ficus-indica* cladode extract (100 mg/kg body weight) was able to reduce the oxidative lithium-induced damage through the increase in antioxidant enzyme levels (superoxide dismutase, catalase, and glutathione peroxidase). This effect is probably associated with the capacity of this extract to reduce the lipid peroxidation level in membrane cells by scavenging free radicals [71]. Moreover, the research conducted by Saad et al. [71] also demonstrates that cladode extract was able to reactivate the erythropoiesis mechanism and thus enhance the production of erythropoietin. Experimental data also suggested a protective effect of ethanolic extract of cladodes found in methotrexate-induced damage in rat intestine [72] and in kidney dysfunction via antioxidant, anti-genotoxic and antiapoptotic properties against cis-diamine dichloroplatinum [73]. Akacha et al. [72] demonstrated that the combined treatment of methotrexate with *O. ficus-indica* extract significantly contributes to reduce the induced oxidative damage of methotrexate that is a chemotherapeutic element for various inflammatory diseases. According to Brahmi et al. [73], *O. ficus-indica* cladodes extracts can inhibit microsomal activation or can directly protect DNA strands from the electrophilic metabolite of the mutagen. In addition, they may inhibit several metabolic intermediates and reactive oxygen species formed during the process of microsomal enzyme activation, which is capable of breaking DNA strands [73]. Tesoriere et al. [74] have shown that 2 weeks of supplementation with 500 g of *O. ficus-indica* fruit pulp decreases lipid peroxidation, demonstrated by a 4-fold decrease of malondialdehyde and about one-third decrease of 8-epi-PGF2α, and improves antioxidant status in healthy individuals [74]. López-Romero et al. [75] found similar results not only in healthy but also in type 2 diabetic patients. Their results suggest that *O. ficus-indica* may also reduce serum insulin, postprandial blood glucose and plasma glucose-dependent insulinotropic peptide peaks [75]. These actions can justify the interest in the prickly pear extracts prepared, using not only the pulp but also cladodes for pharmaceutical use, but also the importance of including the fruit in daily diet. Although seeds and peel have quercetin, no known data addressed its effects on oxidative stress.

### 4.2. Antibacterial Activity

The discovery and use of new antimicrobial agents, mostly from plants, can be an alternative to help overcome antimicrobial resistance, one of the most serious health problems. Ramírez-Moreno et al. [76] tested the antimicrobial activity of the *O. ficus-indica* seeds oil against *Candida albicans*, *Escherichia coli* O58: H21, *Escherichia coli* O157: H7, *Staphylococcus aureus*, *Listeria monocytogenes*, *Pseudomonas aeruginosa*, *Saccharomyces cerevisiae* and *Salmonella typhimurium*. The results showed that the oil extracts have high antimicrobial activity against Gram-positive and Gram-negative bacteria. In another study, developed by Welegerima and Zemene [77], the antimicrobial potential of extracts from the prickly pear peel was studied. In this study, the peel extracts showed greater antimicrobial activity against Gram-positive bacteria than Gram-negative bacteria. The results obtained also demonstrated a greater antimicrobial activity of the prickly pear peel against *S. typhimurium* (S456), *Bacillus subtilis* (B2836) and *Streptococcus pneumoniae* (ATCC63) compared to tetracycline and vancomycin. Cladodes extracts also have antimicrobial activity. According to the results obtained by Welegerima et al. [78], cladodes extracts have antibacterial activity against both Gram-positive (*B. subtilis* and *S. pneumoniae*) and Gram-negative bacteria (*E. coli* and *S. typhimurium*).

### 4.3. Anti-Inflammatory and Antiproliferative Effects

Inflammation is quite complex and has varied responses, having multiple implications in homeostasis. Few data have pointed out possible anti-inflammatory actions of prickly pear components. Gentile et al. [79] studied the effect of betanin and indicaxanthin with an in vitro model of the inflammatory reaction on the expression of endothelial adhesion molecules. With the results obtained, the authors verified that the two phytochemicals slightly inhibited the expression of the cell adhesion molecule ICAM-1. These pigments can modulate the expression of adhesive molecules in endothelial cells, in addition to their antioxidant and free radical scavenging effects [79]. Isorhamnetin diglycosides together with ferulic acid and betacyanins have shown an inhibitory effect on the proliferation of HT-29 colorectal cancer cells. These compounds were extracted from the *O. ficus-indica* byproducts using hydroalcoholic solvents and adsorption separation processes [80]. Naselli et al. [81] studied the effect of the aqueous extract of the *O. ficus-indica* pulp and of its pigment indicaxanthin on the proliferation of human colon cancer Caco-2 cells. The results obtained by the authors indicated that the aqueous extract of *O. ficus-indica* and indicaxanthin had antiproliferative activity in human colon cancer cells and that indicaxanthin may be a modulator of cancer-induced epigenetic variations [81]. Other in vitro studies have shown important antitumoral actions of betalains in the breast, stomach, central nervous system, and lung cell-lines [82]. These substances point out the peel, and not only the pulp, as important parts of the fruit to get most of the present bioactive compounds.

### 4.4. The Influence on Carbohydrate and Lipid Metabolism

Fiber has been for a long time an important element in glycemic control and fat absorption due to its physiologic actions (e.g., gastric emptying), but more recently, other food components have deserved further attention in this process. In what refers to carbohydrate metabolism, polyphenols have shown some interesting actions in carbohydrate digestion and glucose uptake, improving glucose homeostasis [83,84]. Padilla-Camberos et al. [85] studied the hypocholesterolemic activity of the aqueous extract of *O. ficus-indica* in triton-induced mice. They also evaluated the inhibitory activity on the pancreatic lipase enzyme, in vitro, by the same extract. The results showed that aqueous extracts of *O. ficus-indica* could inhibit the enzymatic function of pancreatic lipase, preventing hypercholesterolemia, in part due to its polyphenolic compounds. In addition to these data, prickly pear seeds are important sources of monounsaturated and polyunsaturated fatty acids, which may be responsible for these effects. An experimental study has proven an improving effect of seed oil in glucose levels [86,87]. Berraaouan et al. [86] and Ennouri et al. [87] have also shown that these extracts are able to decrease cholesterol levels. The fatty acid profile, including linoleic and oleic acids present in seeds but also in the peel, can justify this effect.

### 4.5. Neuroprotective Effects

The neuroprotective effects from *O. ficus-indica* may be due to actions attributed to the compounds present in the pulp of the fruit, but also seeds and possibly cladodes [88]. The most frequently pointed out mechanisms that can cause neuronal damage are related to inflammation and oxidative stress. Thus, multiple components present in *O. ficus-indica* can prevent these pejorative phenomena and protect the brain and nervous system. Kim et al. [89] studied the neuroprotective effect of methanolic extracts of *O. ficus-indica* in in vitro and in vivo models of ischemia. The authors have shown that *O. ficus-indica* methanolic extracts are able to reduce excitotoxic damage provoked by ischemia, which is also a possible cause for neuronal damage. The samples were initially subjected to methanol extraction and fractionated successively until a water fraction was obtained. Wie [90] studied the inhibitory action of *O. ficus-indica* methanol extracts on xanthine/xanthine oxidase, FeCl_2_/ascorbic acid and arachidonic acid-induced neurotoxicity in mouse cortical cell cultures. The results obtained have suggested that extracts of *O. ficus-indica* may contribute to neuroprotection in neuronal damage promoted by free radicals or oxygen deprivation. Dok-Go et al. [91] showed that compounds like quercetin, dihydroquercetin and quercetin-3-methyl-ether, present not only in the fruit pulp but also in the cladodes, are able to reduce the damage promoted by hydrogen peroxide, as well as xanthine oxidase in rat cortical cells. The compounds studied were isolated from the ethyl acetate fraction of the fruits and stems of *O. ficus-indica* var. saboten [91]. Considering that the brain is quite susceptible to oxidation due to the richness in unsaturated fatty acids and transition metals (e.g., iron) and the nervous system antioxidant defenses have a low-efficiency, there is a high potential in the antioxidant role of the compounds present in prickly pear. There are no known data from clinical studies due to the difficulties in proving these effects in vivo. Nevertheless, the importance of fresh fruits in supplying components, which have these preventive actions, is well accepted and has been proven.

## 5. Current Applications

The antioxidant actions from the compounds present in *O. ficus-indica* have been of great value in other areas of science, including the food industry. The young cactus cladodes are frequently consumed as vegetables, in salads, while the prickly pear fruit is consumed as a fresh fruit [92]. In Brazil, Chile and Mexico, it is also used for livestock forage [93].

Msaddak et al. [94,95] suggested an interesting effect of adding an extract from *O. ficus-indica* cladodes extract to bread, as well as in cookies. The obtained results showed that rising levels of cladodes powder (2.5 to 10%) contributed to the increase of antioxidant activity of high-fat cookies and decreased their oxidative degradation [95]. On the other hand, it was found that in wheat bread formulation, a substitution of up to 5% of wheat flour by cladodes powder was possible without changing the physical and sensory properties [94].

Cornejo-Villegas et al. [96] studied the physicochemical properties of commercial corn flours added with *O. ficus-indica* powder to achieve the same calcium and fiber contents as the traditional corn flours. The authors were able to obtain final products with fiber and calcium contents like those of traditional corn flours with the inclusion of 4 to 6% of *O. ficus-indica* powder [96].

El Samahy et al. [97] produced a new ice cream using a 5% substitution of cactus pulp concentrate. *O. ficus-indica* can be a good alternative to produce ice creams because of its low acidity, high sweetness, nutritive value and attractive colors [97]. Another study showed that adding an extract from *O. ficus-indica* flowers can improve olive oil stability to oxidative damage [98].

Oniszczuk et al. [99] added 2.5–15% of prickly pear fruits and obtained a gluten-free pasta with good antioxidant properties [99].

## 6. Conclusions

Considering the nutritional composition of prickly pear byproducts, particularly in terms of minerals and unsaturated fatty acids, it is possible to conclude that byproducts can be industrially exploited. According to our review, there is a realistic potential of *O. ficus-indica* byproducts to be used in the development of functional foods and/or to extract added value compounds that can be further applied in food, cosmetic and pharmaceutical industries. To our best knowledge, most of these byproducts are discarded as waste, and they are valuable sources not only of minor compounds (vitamins and minerals) but also fiber and fatty acids. Moreover, concerning the literature, most of the aforementioned compounds have already been used, and potential human health effects were demonstrated.

The use of byproducts of *O. ficus-indica* is a very important aspect that can contribute to an increase in the sustainable production of different industries (e.g., food, cosmetics) and to a more effective food waste management. The transformation of food byproducts into new raw materials makes it possible to move to a closed economic system. The attempt to implement the concept of the circular economy creates opportunities for the food industry to use its raw materials more efficiently, obtaining value-added food products, and achieving a reduction in environmental impact and sustainable economic growth. In addition to this, we have more modern technologies that are based on the principles of “Green Chemistry” that allow more efficient use of food byproducts.

It is of utmost importance to continue investing in the research and analysis of new bioactive compounds with interesting biological properties and to optimize their extraction techniques, making them more environmentally friendly. It is also particularly important to develop strategies to ensure the viability of the processes used so that they can be implemented at the industrial level and to ensure the valorization of the nutritional and functional potential of *O. ficus-indica* and its byproducts.

## Figures and Tables

**Table 1:** Nutritional composition of *Opuntia ficus-indica* (L.) Mill.

Components	Units	Parts of *O. ficus-indica*				Reference
		Pulp	Seed	Peel	Cladode	
Moisture	g/100 g				94.00 ± 0.78	[10]
		87.07 ± 0.86				[12]
		90.89 ± 0.36				[13]
		94.40 ± 2.61	18.05 ± 2.53	90.33 ± 0.21		[14]
		82.27 ± 2.22				[15]
		90.66 ± 2.32		88.92 ± 1.98		[16]
					90.67 ± 0.75	[17]
					92.33 ± 1.36	[18]
					91.09 ± 0.25	[19]
					94.95 ± 1.39	[20]
				80.17 ± 0.93		[21]
			6.10 ± 0.7			[22]
			5.30 ± 0.51			[23]
		88.59 ± 1.17				[24]
Ash	g/100 g				1.08 ± 0.11	[10]
		4.03 ± 0.52				[12]
		0.32 ± 0.04				[13]
		0.56 ± 0.00	10.37 ± 0.51	0.29 ± 0.01		[14]
		0.39 ± 0.09				[15]
		0.24 ± 0.03		0.38 ± 0.03		[16]
	g/100 g of dry matter	8.50 ± 0.82	5.90 ± 1.25	12.10 ± 1.46		[25]
	g/100 g				2.39 ± 0.09	[17]
					0.50 ± 0.01	[18]
					13.22 ± 0.31	[19]
					21.35 ± 1.86	[20]
				1.60 ± 0.02		[21]
			1.27 ± 0.03			[22]
			2.84 ± 0.15			[23]
	g/100 g of dry matter				14.40 ± 0.38	[26]
					18.57 ± 7.57	[27]
Protein	g/100 g				0.30 ± 0.07	[10]
		1.03 ± 0.01				[12]
		0.18 ± 0.05				[13]
		0.08 ± 0.00	3.67 ± 0.01	0.14 ± 0.01		[14]
		0.90 ± 0.26				[15]
		0.15 ± 0.02		0.17 ± 0.02		[16]
	g/100 g of dry matter	5.13 ± 0.29	11.80 ± 1.17	8.30 ± 0.90		[25]
	g/100 g				0.82 ± 0.05	[17]
					0.58 ± 0.02	[18]
					2.49 ± 0.08	[19]
	g/100 g of dry matter				7.81 ± 0.99	[20]
	g/100 g			0.90 ± 0.03		[21]
			15.72 ± 0.38			[23]
	g/100 g of dry matter				11.20 ± 1.68	[26]
	g/100 g	0.39 ± 0.07				[24]
	g/100 g of dry matter				13.06 ± 4.52	[27]
Crude protein	g/100 g		4.78 ± 0.60			[22]
Fat	g/100 g	0.04 ± 0.00	3.00 ± 0.17	0.10 ± 0.01		[14]
		0.50 ± 0.13				[15]
		0.05 ± 0.00		0.04 ± 0.00		[16]
	g/100 g of dry matter	0.97 ± 0.06	6.77 ± 0.51	2.43 ± 0.32		[25]
	g/100 g				0.37 ± 0.02	[17]
					0.12 ± 0.02	[18]
					1.83 ± 0.36	[20]
			16.29 ± 0.21			[23]
	g/100 g of dry matter				0.91 ± 0.23	[26]
					0.74 ± 0.73	[27]
Crude fat	g/100 g	0.40 ± 0.00				[12]
			5.00 ± 0.90			[22]
Total fiber	g/100 g				2.70 ± 0.41	[10]
		5.37 ± 0.87				[15]
		0.43 ± 0.03		0.65 ± 0.05		[16]
	g/100 g of dry matter	20.50 ± 0.94	54.20 ± 1.06	40.80 ± 1.32		[25]
	g/100 g of total carbohydrates				51.24 ± 2.12	[17]
	g/100 g		46.97 ± 1.38			[23]
	g/100 g of dry matter				15.54 ± 15.43	[27]
Crude fiber	g/100 g	1.37 ± 0.06				[12]
		4.28 ± 1.13				[13]
				0.96 ± 0.06		[21]
			12.47 ± 2.30			[22]
	g/100 g of dry matter				5.97 ± 0.84	[26]
Dietary fiber soluble	g/100 g of total carbohydrates				12.98 ± 0.32	[17]
Dietary fiber insoluble	g/100 g of total carbohydrates				34.58 ± 0.99	[17]
Sugars	g/100 g	11.22 ± 1.47				[13]
	mg dextrose/g				37.93 ± 18.56	[26]
Reducing sugars	g/100 g	10.55 ± 0.69				[13]
					2.87 ± 0.11	[19]
Carbohydrates	g/100 g	92.57 ± 0.99				[12]
					5.63 ± 0.11	[17]
					3.05	[18]
	g/100 g of dry matter				57.87 ± 19.39	[27]
Starch	g/100 g of dry matter	4.55 ± 0.24	5.35 ± 1.14	7.12 ± 0.60		[25]
					0.71 ± 0.10	[17]

Values are expressed as means ± standard deviation or as a mean.

**Table 2:** Mineral content of the different parts of *Opuntia ficus-indica* (L.) Mill.

Minerals	Units	Parts of *O. ficus-indica*				Reference
		Pulp	Seed	Peel	Cladode	
Magnesium	mg/100 g				94.10 ± 28.3	[10]
		8.20				[12]
		1.66 ± 0.35				[13]
		1.05	8.07	1.47		[14]
		25.10 ± 5.70				[15]
		1.74		1.41		[16]
	mg/100 g of dry matter	76.10	208	322		[25]
	mg/100 g				995 ± 102.08	[20]
				195.76		[21]
			11.73 ± 0.11			[22]
			70.84 ± 1.04			[23]
					1380 ± 137	[32]
Sodium	mg/100 g				1.71 ± 0.99	[10]
		2.42				[12]
		1.29 ± 0.30				[13]
		0.06	0.44	0.11		[14]
		0.63 ± 0.82				[15]
		0.07		0.20		[16]
	mg/100 g of dry matter	7.77	<0.83	<0.85		[25]
	mg/100 g				30.83 ± 12.81	[20]
				183.43		[21]
			7.13 ± 0.23			[22]
			64.02 ± 3.31			[23]
					63.33 ± 5.77	[26]
					1918 ± 105	[32]
	mg/100 mL juice	4.65 ± 0.56		5.61 ± 0.12		[33]
Potassium	mg/100 g				224 ± 74	[10]
		14.07				[12]
		17.11 ± 3.31				[13]
		11.14	64.41	9.48		[14]
		158.30 ± 32.80				[15]
		38.36		60.83		[16]
	mg/100 g of dry matter	559	275	3430		[25]
	mg/100 g			63.46		[21]
			53.27 ± 1.17			[22]
			154.36 ± 4.83			[23]
					2403.30 ± 234.38	[26]
					1684 ± 68	[32]
	mg/100 mL juice	21.55 ± 0.94		20.26 ± 1.89		[33]
Calcium	mg/100 g				177 ± 31	[10]
		40.92				[12]
		0.23 ± 0.05				[13]
		0.69	17.37	1.52		[14]
		26.30 ± 7.60				[15]
		4.58		2.93		[16]
	mg/100 g of dry matter	163	258	2090		[25]
	mg/100 g			188.58		[21]
			47.12 ± 1.23			[22]
			15.34 ± 1.23			[23]
					626.67 ± 15.28	[26]
					7518 ± 162	[32]
Manganese	mg/100 g				0.78 ± 0.24	[10]
		4.89				[12]
		0.30 ± 0.16				[15]
		0.10		0.13		[16]
	mg/100 g of dry matter	6.99	<0.83	72.9		[25]
	mg/100 g				10 ± 9.51	[20]
				18.01		[21]
					13.83 ± 5.21	[30]
			0.19 ± 0.03			[22]
Iron	mg/100 g				0.13 ± 0.05	[10]
		3.35				[12]
		0.09 ± 0.01				[13]
		0.20 ± 0.06				[15]
		0.30		0.47		[16]
	mg/100 g of dry matter	16.50	12.10	8.31		[25]
	mg/100 g				13.67 ± 4.76	[20]
				25.58		[21]
			1.17 ± 0.17			[22]
			8.95 ± 1.14			[23]
					8.62 ± 8.14	[26]
	mg/100 mL juice	0.22 ± 0.01		0.15 ± 0.10		[33]
Zinc	mg/100 g				0.37 ± 0.21	[10]
		1.63				[12]
		0.003 ± 0.00				[13]
		0.21 ± 0.05				[15]
		0.07		0.13		[16]
	mg/100 g of dry matter	1.55	4.16	1.70		[25]
	mg/100 g				6.17 ± 2.14	[20]
				17.85		[21]
			0.32 ± 0.03			[22]
			1.37 ± 0.28			[23]
Copper	mg/100 g				0.06 ± 0.03	[10]
		0.001				[12]
		0.004 ± 0.00				[13]
		0.04 ± 0.01				[15]
		0.14		0.19		[16]
	mg/100 g of dry matter	<0.78	<0.83	<0.85		[25]
	mg/100 g			9.48		[21]
			0.21 ± 0.07			[22]
			0.30 ± 0.09			[23]
	mg/100 mL juice	0.22 ± 0.02		0.21 ± 0.02		[33]
Phosphorus	mg/100 g	0.006				[12]
		2.31 ± 0.55				[13]
		0.26		0.53		[16]
	mg/ 100 g of dry matter	0.06	110	0.06		[25]
	mg/100 g		162.75 ± 2.73			[22]
			143.94 ± 4.55			[23]
					0.09 ± 0.00	[26]
Molybdenum	mg/100 g				16.38 ± 6.49	[10]
	mg/ 100 g of dry matter	<0.31	<0.33	<0.34		[25]
Chromium	mg/100 g				0.03 ± 0.01	[10]
		0.01 ± 0.00				[15]
Nickel	mg/100 g	0.03 ± 0.01				[15]

Values are expressed as means ± standard deviation or as a mean.

**Table 3:** Vitamins content of the different parts of *Opuntia ficus-indica* (L.) Mill.

Vitamins	Units	Parts of *O. ficus-indica*					Reference
		Pulp	Pulp Oil	Seed Oil	Peel	Cladode	
Vitamin C	mg/100 g	5.17 ± 0.06					[12]
		33.40 ± 4.53					[45]
	mg ascorbic acid eq/100 g	54.64 ± 10.54			109.69 ± 19.90		[46]
Ascorbic acid	mg/100 g	29 ± 1					[7]
						1.83 ± 0.33	[10]
		17.20 ± 4.43					[15]
					59.82 ± 0.64		[21]
	mg/100 mL	0.03 ± 0.00					[39]
		7.12 ± 1.83					[40]
	mg/100 g	45.8					[41]
		28.67 ± 3.63					[24]
		18.50 ± 2.00					[42]
		21.20 ± 0.573					[43]
		34 ± 12.17					[44]
Total vitamin E	mg/100 g of total lipids				2180 ± 198		[35]
	μg/100 g	115 ± 10					[36]
	mg/100 g		527.40 ± 36.00	40.30 ± 4.00			[37]
α-Tocopherol	mg/100 g of total lipids				1760 ± 155		[35]
	μg/100 g	69 ± 5.90					[36]
	mg/100 g		84.9 ± 9	5.60 ± 0.30			[37]
β-Tocopherol	mg/100 g of total lipids				222 ± 45		[35]
	mg/100 g		12.60 ± 1.00	1.20 ± 0.20			[37]
δ-Tocopherol	mg/100 g of total lipids				26 ± 12		[35]
	μg/100 g	16 ± 1					[36]
	mg/100 g		422 ± 17	0.50 ± 0.10			[37]
γ-Tocopherol	mg/100 g of total lipids				174 ± 31		[35]
	μg/100 g	30 ± 3					[36]
	mg/100 g		7.90 ± 0.60	33 ± 3			[37]
Vitamin K	mg/100 g of total lipids				109 ± 32		[35]
	mg/100 g		53.20 ± 8.00	525 ± 60			[37]

Eq—equivalents. Values are expressed as means ± standard deviation or as a mean.

**Table 4:** Fatty acids content of the different parts of *Opuntia ficus-indica* (L.) Mill.

Fatty Acids						Parts of *O. ficus-indica*							
	Pulp Oil	Pulp	Seed Oil					Peel			Cladode		
Lauric		1.55 ^b^						0.71 ± 0.15 ^d^		0.08 ^b^	1.33 ^e^	0.91 ^b^	
Myristic	1.13 ± 0.09 ^a^	1.52 ^b^				0.13 ± 0.02 ^c^		1.95 ± 0.25 ^d^		0.30 ^b^	1.96 ^e^	1.88 ^b^	
Palmitic	34.4 ± 3.12 ^a^	22.9 ^b^	20.1 ± 2.26 ^a^	9.32 ± 0.19 ^b^	11.66 ± 0.48 ^c^	12.2 ± 1.70 ^c^	13.2 ± 0.33 ^c^	23.1 ± 1.98 ^d^	23.7 ^c^	27.0 ^b^	13.9 ^e^	39.0 ^b^	19.1 ^b^
Stearic	2.37 ± 0.10 ^a^	5.20 ^b^	2.72 ± 0.13 ^a^	3.11 ± 0.04 ^b^	3.93 ± 0.49 ^c^	0.15 ± 0.03 ^c^	3.65 ± 0.39 ^c^	2.67 ± 0.21 ^d^	3.93 ^c^	2.23 ^b^	3.33 ^e^	5.57 ^b^	4.82 ^b^
Arachidonic						0.95 ± 0.07 ^c^			5.52 ^c^				
Palmitoleic	1.62 ± 0.06 ^a^		1.80 ± 0.11 ^a^	1.42 ± 0.01 ^b^				2.48 ± 0.22 ^d^	2.46 ^c^	0.36 ^b^	0.24 ^e^	2.00 ^b^	0.06 ^b^
Oleic	10.8 ± 0.98 ^a^	26.8 ^b^	18.3 ± 1.58 ^a^	16.8 ± 0.47 ^b^	16.56 ± 2.35 ^c^	25.5 ± 1.10 ^c^	19.2 ± 4.22 ^c^	21.1 ± 2.15 ^d^	19.7 ^c^	14.4 ^b^	11.1 ^e^	18.0 ^b^	19.3 ^b^
Linoleic	37.0 ± 3.87 ^a^	29.2 ^b^	53.5 ± 4.89 ^a^	70.3 ± 0.60 ^b^	59.12 ± 2.74 ^c^	61.0 ± 1.30 ^c^		32.3 ± 2.14 ^d^	29.0 ^c^	41.2 ^b^	34.9 ^e^	20.4 ^b^	35.6 ^b^
Linolenic	12.0 ± 1.05 ^a^	12.2 ^b^	2.58 ± 0.16 ^a^					0.69 ± 0.06 ^d^	15.7 ^c^	13.9 ^b^	32.8 ^e^	10.9 ^b^	13.9 ^b^
γ-Linolenic							61.4 ± 1.90 ^c^	8.60 ± 1.04 ^d^			0.40 ^e^		
Behenic								0.50 ± 0.05 ^d^					
Lignoceric								0.41 ± 0.04 ^d^					
*cis*-13,16-Docosadienoic								0.93 ± 0.08 ^d^					
Cerotic								0.35 ± 0.04 ^d^					
Nervonic								1.21 ± 0.26 ^d^					
Reference	[37]	[47]	[37]	[51]	[52]	[22]	[49]	[35]	[21]	[47]	[48]	[47]	[47]

Values are expressed as means ± standard deviation or as a mean. ^a^ expressed as g/100 g of oil. ^b^ expressed as g/100 g of total fatty acid. ^c^ expressed as g/100 g. ^d^ expressed as g/100 g of total fat. ^e^ expressed as g/100 g of fatty acid methyl esters.

**Table 5:** Fatty acid profile (g/100 g) of seeds oils [55].

Oil	Mango Seed	Apricot Kernel	Paprika Seed	Blackcurrant Seed	Watermelon Seed	Pumpkin Seed	Cranberry Seed	Grape Seed	Prickly Pear
Total SFA	52.8	4.8	17.6	8.3	21.7	22	9.7	6.7	30.4
Total MUFA	41.3	66.4	14.7	16.3	18.4	26.1	22.7	18.4	16.3
Total PUFA	7.4	28.8	67.8	75.3	60	51.5	67.6	65.4	52.9
Linoleic acid	6.9	28.6	67.8	61.5	59.6	51.3	45.3	64.9	45.6
Linolenic acid	0.5	0.1		13.8	0.4	0.2	22.3	0.6	7.3

SFA, saturated fatty acids; MUFA, monounsaturated fatty acids; PUFA, polyunsaturated fatty acids.

**Table 6:** Amino acid content of the different parts of *Opuntia ficus-indica* (L.) Mill.

Amino Acids	Parts of *O. ficus-indica*				
	Pulp			Seed Meal	Cladode
Taurine	ND	9.36 ± 2.04	323.60 ± 572.10		
Aspartate	844.24 ± 256.48			8.60	0.61 ± 0.06
Threonine	120.71 ± 48.48		13.10 ± 1.80	3.96	1.38 ± 0.15
Serine	967.94 ± 453.75		175.50 ± 43.46	4.14	0.48 ± 0.11
Asparagine	253.21 ± 102.22		41.60 ± 5.67		
Glutamate	154.25 ± 114.51		66.10 ± 21.06	20.27	1.78 ± 0.32
Glutamine	1583.19 ± 922.78		346.17 ± 205.84		
Proline	6461.79 ± 2476.41		1265.20 ± 455.02	5.66	0.45 ± 0.12
Glycine	174.78 ± 98.60		11.33 ± 4.23	7.67	0.36 ± 0.07
Alanine	353.86 ± 156.29		87.23 ± 8.16	4.58	0.46 ± 0.05
Citrulline	79.14 ± 71.47		16.27 ± 11.54		
Valine	254.38 ± 107.03		39.37 ± 9.28	5.69	0.58 ± 0.09
Cysteine	10.63 ± 8.17			3.10	
Methionine	189.69 ± 83.08		55.23 ± 22.22	2.61	0.15 ± 0.03
Isoleucine	241.39 ± 128.77		31.17 ± 8.63	3.66	0.67 ± 0.05
Leucine	218.72 ± 97.02		20.60 ± 0.99	6.90	0.76 ± 0.12
Tyrosine	208.35 ± 124.69		12.30 ± 2.43	3.56	0.21 ± 0.09
Phenylalanine	337.75 ± 145.43		23.33 ± 1.16	4.46	1.37 ± 0.28
β-Alanine	42.25 ± 21.12				
γ-Aminobutyric acid	2272.69 ± 1181.82				
Histidine	505.48 ± 203.07		45.20 ± 11.29	2.46	0.15 ± 0.03
Tryptophan	117.36 ± 46.55		12.63 ± 4.33	0.90	0.16 ± 0.02
Ornithine	8.37 ± 4.66				
Lysine	205.46 ± 103.14		17.40 ± 1.27	3.39	0.53 ± 0.08
2-Aminoethanolamine	117.31 ± 42.09				
Arginine	375.87 ± 272.37		30.50 ± 8.29	14.62	0.16 ± 0.02
Carnosine			5.90 ± 1.71		
Reference	[56]	[36]	[57]	[23]	[20]
Units	µmol/L	mg/100 g	mg/L	g amino acid/100 g protein	g/100 g protein

ND—not detected. Values are expressed as means ± standard deviation or as a mean.

## Data Availability

Data sharing not applicable.
